# Examining the Emergency Department Care Experiences of Equity-Deserving Groups Using an Intersectional Lens

**DOI:** 10.1177/21501319241290888

**Published:** 2024-10-21

**Authors:** Aliki Karanikas, Reyana Jayawardena, Maya Balamurugan, Susan A. Bartels, Melanie Walker

**Affiliations:** 1 Faculty of Health Sciences, Queen’s University, Kingston, ON, Canada; 2 Department of Emergency Medicine, Queen’s University, Kingston, ON, Canada; 3 Department of Public Health Sciences, Queen’s University, Kingston, ON, Canada

**Keywords:** emergency department, intersectionality, social determinants of health, health equity, vulnerable populations, mental health, people who use substances, patient-centeredness, underserved communities

## Abstract

**Introduction:**

Equity-deserving groups (EDGs) face societal barriers, including healthcare barriers within the emergency department (ED), due to discrimination. Most patient-care experience research considers only a single-axis perspective, neglecting multifaceted impacts of discrimination, or intersectionality.

**Methods:**

Detailed is a secondary analysis of a mixed-methods, cross-sectional study conducted at the Kingston Health Sciences Centre (KHSC) between June and August 2021. A quantitative analysis was conducted to identify differences between participants who did not identify as equity-deserving (controls), and those who identified with 1, 2, or 3 EDGs, respectively. The research team conducted thematic analysis on the shared micronarratives to contextualize the quantitative results. The research team also held focus groups with community partners that served EDGs to gain their insights on study findings and add their perspectives to the captured themes.

**Results:**

Comparing 1973 individuals belonging to none, 1, 2, or 3 EDGs revealed significant differences in patient-perceived attention to their needs (*P* < .001), patient-control in health care decision-making (*P* = .001), and whether quality medical care or experiencing kindness/respect was more important (*P* = .003). Three themes were identified: *stigma and discrimination*, *lack of patient-centered care*, and *need for improved patient-provider communication*.

**Conclusion:**

The study’s findings contribute to a sparse body of evidence on EDG-care experiences in the ED through an intersectionality lens. Future research efforts should evaluate the complex interactions of specific EDG memberships to improve care experiences.

## Introduction

The emergency department (ED) is a unique healthcare setting that sits at the border of ambulatory services and hospital-based care.^1^ The ED often serves as a safety net for diverse groups of people.^1^ Equity-deserving groups encompass individuals and populations who have historically faced barriers to opportunities, care access, and resources due to systemic disadvantage and colonialism.^1,2^ These groups include Indigenous people, the 2SLGBTQ+ community, individuals with mental health concerns, the vulnerably housed, and people who use substances (PWUS), to name a few.^1,2^ Studies have shown that EDGs often use the ED at a higher rate than their non-EDG counterparts because of service accessibility challenges and difficulties with obtaining primary care.^1,3-7^ Similarly, EDGs are more likely to perceive their ED experience as negative, feel disrespected and/or judged, and feel that their identity impacts the care they receive.^2-6,8-20^ Thus, adopting an intersectional approach to ED care may allow for improvements in the healthcare experiences of EDGs.^5,21^

The term “intersectionality” was first introduced by Kimberlé Crenshaw within her work in antidiscrimination law, where she theorized that the intersection of multiple social identities can converge to create unique forms of oppression.^22,23^ Intersectionality sheds light on the interaction of oppressive systems which shape one’s social position, lived experiences, and interactions with others.

Gaining insights on the ED experiences of EDGs from an intersectional lens is vital to better understand the overlap of oppressed identities among these groups.^17^ For instance, individuals often report increased stigma for identifying with both using substances and experiencing homelessness, highlighting the 2-way relationship among these vulnerabilities.^17^ Similarly, a previous study involving patients presenting with a mental health concern in the ED identified that 30% of participants engaged in harmful alcohol use, 28% were affected by drug use, and 13% experienced both drug and alcohol-related disorders.^18^ Lastly, 2 studies found that amongst those identifying as unhoused, also being Indigenous predicted a higher utilization of ED services.^10,11^

Previous literature has not captured the breadth of patient care experiences from individuals at the intersection of multiple EDGs. Thus, most studies fail to consider the influence that belonging to multiple EDGs may have on patients’ care and interactions with health care providers (HCPs).^3,24,25^ Additionally, past studies on the ED care experiences of EDGs have primarily used qualitative methodology.^9,12,16,17,26,27^ We decided upon a mixed methods study design in order to take a more holistic approach and incorporate the rich data collected from both micronarratives and survey responses. Therefore, this study aims to (a) utilize the frame of intersectionality to evaluate the association between EDG membership and overall ED care experiences, and (b) elucidate themes from shared qualitative data to further contextualize quantitative findings and identify methods to improve ED care.

## Methods

This study was conducted as a secondary analysis of a participatory, mixed-methods, cross-sectional parent study that set out to better understand the ED care experiences of historically marginalized patients. Data collection for the parent study took place in the ED of Kingston General Hospital (KGH) and the Urgent Care Centre (UCC) of Hotel Dieu Hospital (HDH) in Kingston, Ontario, Canada between June and August 2021. The data was collected through an open-ended audio recording prompt as well as follow up survey questions. Community partners that support EDGs were also able to provide clients with the same survey to share a previous experience as they might have no longer been seeking ED care due to negative experiences.

A convenience sample of study participants was included with individuals 16 years of age or older who had visited the KGH ED or HDH UCC within the previous 24 months, as either the patient themselves or accompanying someone else. Participants were able to share either first person or third person experiences (termed micronarratives). Individuals were ineligible if they were medically unstable, aggressive toward staff and/or could not provide informed consent. Participants identified the patient in the micronarrative as either identifying with no EDG (control) or identifying with up to 3 EDGs.

**Figure A1.**
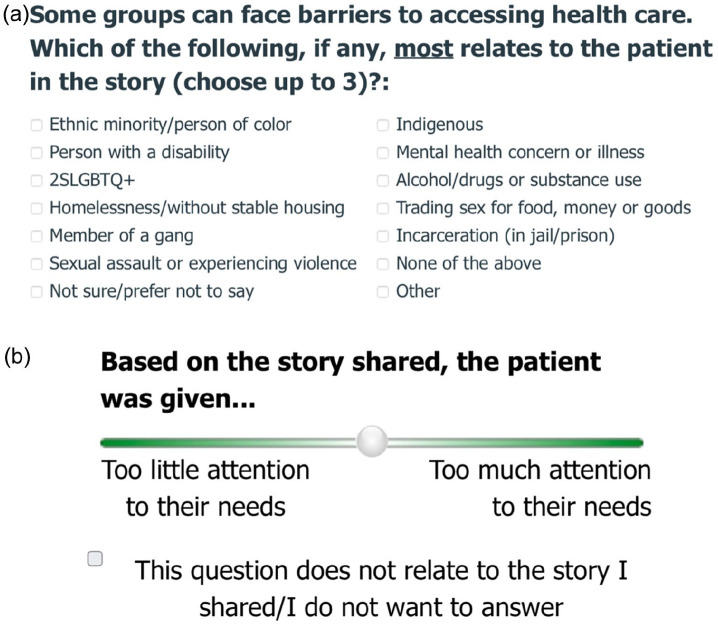
(a) Survey question EDG membership and ED experience and (b) example slider question.

All data were collected in English on the Spryng.io app on handheld tablets.^28^ Participants were asked to share an ED care experience from the past 24 months in response to one of the open-ended prompting questions and then self-interpreted their experiences by answering pre-defined survey questions, including those with 3 answer choices (triads), 2 answer choices (sliders), and multiple-choice questions (MCQs). MCQs also collected participant demographics and ED visit characteristics. In the MCQs, participants were able to select up to 3 EDGs that they felt related most to the experience of the patient in the story, if applicable. Participants could identify with historically marginalized groups in the demographic section without necessarily selecting that EDG membership was related to the story shared. The Figure A1 in the Appendix provides an example of both a slider question and the MCQ pertaining to EDG membership.

Descriptive statistics of participant demographics and ED visit characteristics were first conducted in SPSS (IBMM SPSS Statistics Version 28.01.1). Chi squared tests evaluated differences in characteristics amongst participants who identified with none (controls), 1, 2, or 3 EDGs. The slider responses were graphically represented as histograms using Tableau (Version 2022.3.0), and the area under the bars were collectively analyzed using a Kruskal-Wallis *H* Test in SPSS. This test was used to determine if the differences between groups were statistically significant.^30^ *P* values of <.05 for both the chi-squared tests and the Kruskal-Wallis *H* Tests were considered statistically significant.

The research team conducted an inductive and deductive thematic analysis to contextualize the quantitative findings. The deductive analysis was based on a codebook developed by study personnel who took into consideration the parent study survey questions and existing literature. The inductive coding aspect was the result of emergent themes from participant micronarratives.^29^ The first-person micronarratives shared by participants who identified with 3 EDGs and indicated that their experience was about a lack of respect/judgment were selected for qualitative analysis. These micronarratives were selected as they were most likely to elucidate the positive and negative care experiences in the ED and thereby contextualize the quantitative findings. This approach also assisted with study feasibility as a result of time constraints. Reviewers met to discuss coding and resolve discrepancies. Qualitative analysis was conducted using NVivo 12, and overarching themes were then identified.

Following the mixed methods analysis, 7 focus groups were hosted in 2022/2023 involving community members who identified with various EDGs. Participants were presented with a summary of study findings and asked whether they resonated with their lived experiences and if anything was missing in the captured data. Focus group participants were also asked to provide ED care suggestions for EDGs.

## Results

There were a total of 2114 surveys completed in the parent study from 1973 unique participants. Of the 1973 unique participants, 949 identified as controls while 994 identified as equity deserving. In total, 1740 participants (82.3%) shared a first-person narrative while 154 (7.3%) shared a third person narrative arising from accompanying someone else to the ED. Within the EDGs, 503 (50.6%) self-identified with 1 EDG, 223 (22.4%) self-identified with 2 EDGs, and 268 (27.0%) self-identified with 3 EDGs. Please see Table 1 for further participant details. Differences between controls and participants identifying with 1, 2, or 3 EDGs were observed including difficulty making ends meet (*P* < .001), ED visit frequency (*P* < .01), sharing an experience about a lack of respect or judgment (*P* < .0001), and feeling that personal situation, identity, or culture impacted care (*P* < .0001).

**Table 1.** Characteristics of Study Participants and ED Visits.^I,II^

Demographic characteristics	Controls n = 949 (48.1%)	Participants identifying with 1 EDG n = 503 (25.5%)	Participants identifying with 2 EDGs n = 223 (11.3%)	Participants identifying with 3 EDGs n = 268 (13.6%)	*P*-values^III^
Age (years)					*P* = .02
<18	67 (7.1%)	31 (6.2%)	15 (6.7%)	12 (4.5%)	
18-25	107 (11.3%)	55 (10.9%)	24 (10.8%)	37 (13.8%)	
26-45	185 (19.5%)	112 (22.3%)	43 (19.3%)	76 (28.4%)	
46-65	175 (18.43%)	90 (17.9%)	43 (19.3%)	57 (21.3%)	
>65	144 (15.17%)	73 (14.5%)	21 (9.4%)	20 (7.5%)	
Missing	271 (28.6%)	142 (28.2%)	77 (34.5%)	66 (24.6%)	
Total	949	503	223	268	
Gender identity					*P* = .35
Man	379 (39.9%)	215 (43%)	104 (46.6%)	113 (42.2%)	
Woman	532 (56.1%)	262 (52.1%)	108 (48.4%)	135 (50.4%)	
Non-binary	11 (1.2%)	10 (2%)	4 (1.8%)	5 (1.9%)	
Missing	27 (2.8%)	16 (3.2%)	7 (3.1%)	15 (5.6%)	
Total	949	503	223	268	
Sexual orientation					*P* = .03
Gay/Lesbian	33 (3.5%)	11 (2.2%)	8 (3.6%)	7 (2.6%)	
Bisexual	57 (6%)	35 (7%)	15 (6.7%)	30 (11.2%)	
Straight	755 (79.7%)	394 (78.3%)	172 (77.1%)	204 (76.1%)	
Pansexual	12 (1.3%)	14 (2.8%)	6 (2.7%)	8 (3%)	
Other^IV^	18 (1.9%)	4 (0.8%)	8 (3.6%)	4 (1.5%)	
Missing	74 (7.8%)	45 (8.9%)	14 (6.3%)	18 (6.7%)	
Total	949	503	223	271	
Ethnicity					*P* = .05
Black	13 (1.4%)	6 (1.2%)	1 (0.4%)	5 (1.9%)	
Indigenous	35 (3.7%)	23 (4.6%)	14 (6.3%)	23 (8.6%)	
1 or more ethnicity	14 (1.5%)	11 (2.2%)	8 (3.6%)	5 (1.9%)	
White/European	549 (57.8%)	276 (54.9%)	113 (50.7%)	155 (57.8%)	
Other^V^	45 (4.7%)	32 (6.4%)	8 (3.6%)	12 (4.5%)	
Missing	293 (30.8%)	155 (30.8%)	79 (35.4%)	68 (25.4%)	
Total	949	503	223	268	
Disability					*P* < .00001
Mental health	74 (7.8%)	49 (9.7%)	24 (10.8%)	62 (23.1%)	
Physical	73 (7.7%)	45 (8.9%)	23 (10.3%)	21 (7.8%)	
Patient is not a person with a disability	430 (45.3%)	213 (42.3%)	75 (33.6%)	95 (35.4%)	
Other^VI^	52 (5.5%)	34 (6.8%)	12 (5.4%)	11 (4.1%)	
Missing	320 (33.7%)	162 (32.2%)	89 (39.8%)	79 (29.5%)	
Total	949	503	223	268	
How often does patient struggle to make ends meet					*P* < .00001
All the time	65 (6.8%)	44 (8.7%)	38 (17%)	72 (26.9%)	
Often	65 (6.8%)	39 (7.8%)	9 (4%)	33 (12.3%)	
Sometimes	143 (15.1%)	66 (13.1%)	48 (21.5%)	45 (16.8%)	
Rarely	163 (17.2%)	79 (15.7%)	31 (13.9%)	27 (10.1%)	
Never	432 (45.5%)	222 (44.1%)	82 (36.8%)	73 (27.2%)	
Missing	81 (8.5%)	53 (10.5%)	15 (6.7%)	18 (6.7%)	
Total	949	503	223	268	
ED visit frequency					*P* = .007
Did not access care in the emergency room before the experience described	173 (18.2%)	82 (16.3%)	38 (17.0%)	36 (13.4%)	
1-3 times	312 (32.9%)	177 (35.2%)	63 (28.3%)	87 (32.5%)	
4-6 times	67 (7.1%)	37 (7.4%)	14 (6.3%)	35 (13.1%)	
7+ times	34 (3.6%)	17 (3.4%)	7 (3.1%)	21 (7.8%)	
Missing	363 (38.3%)	190 (38.0%)	101 (45.3%)	89 (33.2%)	
Total	949	503	223	268	
Was the shared experience about a lack of respect or judgment					*P* = .00001
No	710 (74.9%)	357 (71%)	158 (71%)	165 (61.6%)	
Yes	148 (15.6%)	99 (19.7%)	47 (21.2%)	78 (29.1%)	
Missing	91 (9.6%)	47 (9.3%)	18 (8.1%)	25 (9.3%)	
Total	949	503	223 (11.3%)	268	
How did personal situation/identity/culture impact care experiences					*P* < .00001
In a bad/very bad way	79 (8.3%)	68 (13.5%)	42 (18.8%)	74 (27.6%)	
It did not impact care	659 (69.5%)	326 (64.8%)	135 (60.5%)	139 (51.9%)	
In a good/very good way	88 (9.3%)	43 (8.5%)	17 (7.6%)	24 (9%)	
Missing	123 (13%)	66 (13.1%)	29 (13%)	31 (11.6%)	
Total	949	503	223	268	

**Figure 1.**
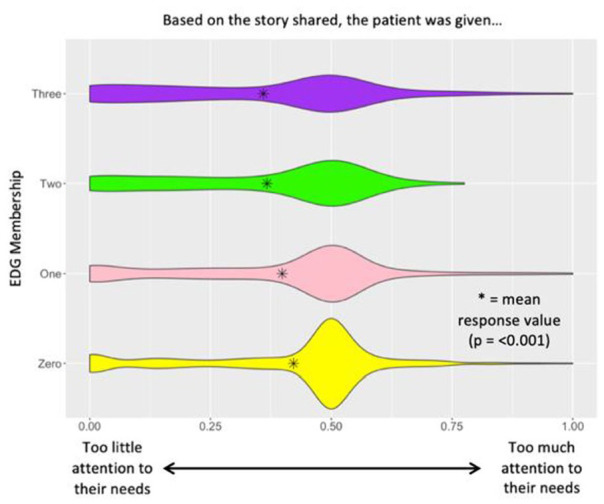
Attention to needs slider question analysis.

**Figure 2.**
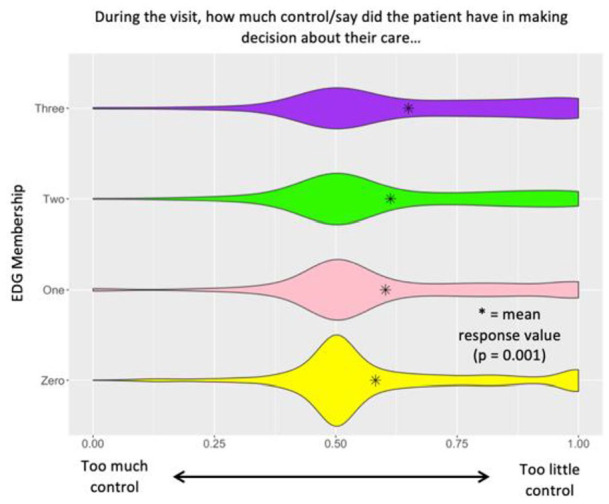
Control/say in decision making slider question analysis.

**Figure 3.**
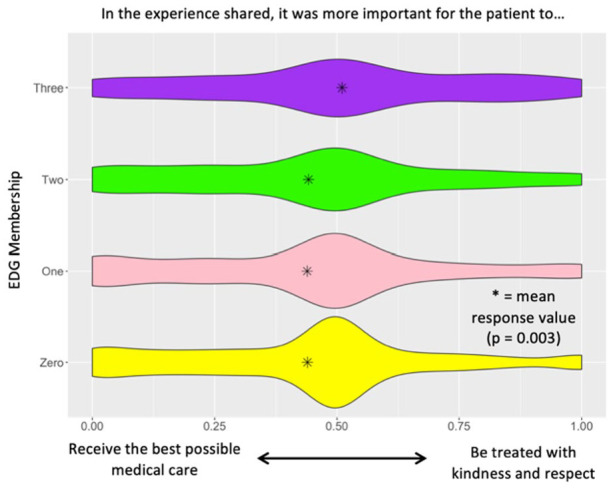
More important aspect of patient visit question slider analysis.

When comparing individuals who belonged to the control group, 1, 2, or 3 EDGs, a statistically significant difference was observed in how much attention patients felt was being given to their needs: *P* < .001 (Figure 1), how much control/say patients felt they had in decision making about their care: *P* = .001 (Figure 2), and whether receiving the best medical care or being treated with kindness and respect was more important for patient visits: *P* = .003 (Figure 3). In Figure 1, it is observed that with increasing EDG membership participants reported that too little attention was being given to their needs. In Figure 2, it is observed that with increasing EDG membership, participants felt they had too little control in making decisions about their care. Lastly, in Figure 3, participants who identified with 3 EDGs responded that it was more important to be treated with kindness and respect than to receive the best medical care possible.

There was a total of 104 micronarratives coded to contextualize the quantitative findings. The first reviewer coded all eligible micronarratives (AK) and a second reviewer (MW) independently coded a random sample (20%) of these micronarratives as a validity check.^31^ Through this analysis, we identified 3 overarching themes: *stigma and discrimination*, *patient-centered care*, and *communication within the ED*, which are presented in Table 2.

**Table 2.** Summary of Themes, Codes, and Further Example Quotes.

Theme	Codes	Example quotes
Stigma and discrimination	Staff attention to personal situation, identity, and culture; feeling judged; feeling respected/lack of respect; feeling accepted/valued or feeling unaccepted/devalued	*The last couple times I went to the emergency room was by ambulance because of overdoses. If they know that you are a user they treat you like shit and act like they are better than you. They talk down to you like you are nothing. Like a dirt bag. It makes me not want to go there. They have to learn how to treat people. Some of us have drug problems. Security is called on me for no reason at all. I’d rather help people than hurt them.* -Patient who self-identified as Indigenous, using substances, and having mental health concerns
Patient-centered care	Staff knowledge of personal situation, identity, and culture, taking patients issues seriously, feeling frustrated, feeling ignored, and feeling dismissed.	*I went to the er room as advised by the detox facility to get potential withdrawal medicine, I followed their advice and went. I spent 4 hours waiting for a doctor just for him to say no to any helpful medication and he gave me Gravol and Suboxone not even knowing my actual dose. I was quite disappointed in my visit to emergency.* -Patient who self-identified as being homeless, using substances, and having mental health concerns
Communication	Communication between staff; communication between patient and staff; support coping with health concerns; and feeling informed/uninformed	*I went in with the paramedics to solve my stomachs. They had residential nurses, I was hallucinating but the staff were not nice. They seem like having miscommunication and constantly arguing about my situation* -Patient who self-identified as experiencing sexual assault, being homeless, and having mental health concerns

A total of 49 participants across 7 focus groups shared their experiences and many participants identified as belonging to more than 1 EDG throughout the discussions. Individuals noted feelings of stigma and discrimination, which corroborated study findings. Participants also discussed the assumptions HCPs would make regarding different EDG memberships. Participants recommended that ED HCPs focus on better listening to equity-deserving patients. It was also suggested that medical students engage in community-based learning to understand the lived experiences of EDGs and gain familiarity with community resources.

## Discussion

This research contributes to the evidence base regarding ED care experiences among EDGs and among those who self-identify with more than 1 EDG, for which literature is limited. The quantitative findings highlight the differences in feelings participants shared about control in decision making, and how much attention participants felt was being given to their medical needs. These results indicate a statistically significant difference between at least 2 levels of EDG membership. These findings also highlight that for those who identified with at least 3 EDGs, it was more important to be treated with kindness and respect. Based on the survey responses indicating statistically significant differences, along with themes that emerged through both the micronarrative analysis and focus groups, it appears that differences between levels of EDG membership were more related to attitudinal and behavioral concerns in provider interactions than to hospital processes or structures. These findings were consistent with the parent study findings where the comparison was between individuals who identified as belonging to an EDG versus identifying as non-EDG participants.^3^ The thematic analysis helped to contextualize quantitative findings by underlining stigmatizing attitudes and the lack of attention given to medical needs reported by participants identifying with 3 EDGs. In a study by Varcoe et al,^2^ some reasons participants believed they were being discriminated against in the ED had to do with belonging to at least 1 EDG. This finding indicates how crucial it is for patients to be treated with inclusivity, especially as this study demonstrated that those identifying with 3 EDGs prefer respectful care over the best possible medical care. Moreover, findings outlined the importance of communication within the ED.

Feelings of judgment, stigma, and discrimination against members of EDGs in the ED are well characterized in the literature, although most studies considered these experiences from a single-axis lens.^8,14,16–20,29,33,34^ In support of our qualitative findings regarding care experiences of individuals with greater numbers of EDG membership, a study by McCallum et al^26^ revealed that individuals reported compounding stigma as a result of identifying with both substance use and experiences of homelessness. The qualitative findings of our study also demonstrated that these feelings of stigma were experienced by individuals who self-identified with 3 EDGs. As well, much of the previous literature identified avoidance of the ED on the part of EDGs because of prior negative care experiences as a reality for these populations.^4,5,8,9,13,15-19,24,27,32-34^ Our qualitative results were consistent with these findings, which could be implicated in worse health outcomes and requires further study. Although our qualitative results focused on individuals belonging to 3 EDGs, similar themes have been identified in previous literature for individuals belonging to other numbers of EDGs. Therefore, these themes are not exclusive to those with membership in 3 EDGs but may be more prominent in their experiences due to the intersection of multiple EDG memberships.

Various recommendations for improving ED equity have emerged in recent years. This study informs the value of applying an intersectional lens to clinical practice within the ED. Intersectionality can help ED HCPs understand the nuanced ways in which a patient’s identities and societal structure can shape their medical experience and treatment preferences, without making assumptions or stereotyping.^21^ Instead, taking an intersectional lens to ED practice involves expanding understanding of the influences affecting a patient’s response to illness and treatments so as to provide patient-centered care.^21^ It is recommended that ED HCPs reflect on their positionality in order to confront their biases and improve the patient-provider relationship.

Moreover, bolstering medical education in terms of how to more compassionately care for members of EDGs, especially those with intersectional identities, could be useful in combatting the negative attitudes and stigma that can be propagated against members from vulnerable communities.^14,35^ Another study suggested the involvement of equity-deserving individuals and their caregivers/support systems within educational initiatives to make the experience more meaningful, such that healthcare HCPs can learn from lived experiences.^36,37^ There still, however, remains a paucity of evidence on outcomes of these recommendations in the literature. As we move forward with future research initiatives, we need to be aware of the “minority tax,” where individuals from marginalized groups are made responsible for promoting equity. It is crucial that research advances with the understanding that the responsibility for equitable health outcomes should be shared by everyone.^38^ Lastly, ensuring undergraduate and postgraduate medical curriculum has a focus on cultural safety could also benefit HCPs, patients, and equity-deserving communities.^35,39^

In our data, there was significant intersectionality between mental health concerns and all other EDGs. Developing ED interventions to better support individuals with mental health concerns, such as training all staff in de-escalation techniques, streamlining the suicide risk assessment process, and employing dedicated and trained psychiatric and social support staff have been effective in improving patient experiences in the ED.^14^

This study is not without its limitations. One such limitation is that the data collection excluded certain demographics of individuals, such as those who were medically unstable or did not speak English, leading to a selection bias. Moreover, because the research assistants were collecting data during select times, there were patient groups that might have been excluded. Additionally, because of a software update issue in the initial stages of data collection, a number of sociodemographic responses on MCQs were not recorded, and led to a large percentage of missing data, albeit random. Although previous literature has explored this area, our study did not specifically evaluate the micronarratives of individuals who related their ED story to membership in 1 or 2 EDGs. While the themes we identified could likely be elucidated from those experiences, they were not explicitly evaluated in our study, presenting a future research opportunity. Lastly, because participants were able to share an ED experience that occurred within the past 24 months, there was a potential for recall bias.

The study also has several strengths. The mixed methods approach allows for the interpretation of quantitative patient reported health outcomes to be supported by the qualitative findings and provide a more holistic analysis of participant experiences within the ED.^40^ As well, the use of the Sensemaking platform reduces social desirability bias, interviewer bias, and empowered participants to interpret their own experiences.^28^ To our knowledge, this is the first study to look at different levels of intersectionality compared with control participants not identifying with any EDGs within the ED setting, while allowing for participants to self-identify with a diverse amount of EDGs. Lastly, recruiting participants not only from 2 healthcare centers (KGH and HDH) but also through community partners allowed for a more comprehensive examination of patient experiences in our region.

## Conclusion

The current study contributes to the body of literature on how members of EDGs perceive care in the ED by considering their experiences based on the various positions of privilege and marginalization they might hold. Our quantitative findings highlighted differences in experiences across the ED encounter between control participants not identifying with any EDGs, and individuals who identified with 1, 2, or 3 EDGs. Thematic analysis contextualized these findings by highlighting feelings of discrimination and stigma from EDG participants, lack of attention to or knowledge of patient needs, and the importance of communication in empowering patients. Further work should consider more granular differences in care experiences based on unique intersections between multiple EDGs. Recommendations to improve these care experiences include having HCPs and trainees grow in their allyship skills, get involved with community partners that support EDGs, and undergo cultural sensitivity training.

## Notes

I. Groups were collapsed when N was <5 based on literature and study personnel discussion. In instances where this was not possible, cell sizes of ≤5 remained to most accurately represent the sociodemographic characteristics respondents identified with. Note that all data collected was anonymous from study start.

II. Participants who selected “Not sure/Prefer not to say” had their responses recoded as “Missing” for the purpose of running descriptive statistics and were omitted from the descriptive analysis.

III. *P*-values less than .05 suggest that there is a significant difference in demographic characteristics (such as age or sex) between at least 2 of the groups.

IV. Other sexual orientations included: asexual, questioning/unsure, and sexual orientation not on the list.

V. Other ethnicities included: Arab, Chinese, Filipino, Japanese, Korean, Latin American, South Asian (Indian, Pakistani. . .), Southeast Asian (Vietnamese, Thai. . .), and West Asian (Iranian/Afghan)

VI. Other disabilities included: hearing loss/deafness, intellectual disability (need help with transportation, managing money. . .), low vision/blindness, and other (learning disability, autism spectrum disorder, neurodiverse. . .)
